# Myoglobin clearance by super high-flux hemofiltration in a case of severe rhabdomyolysis: a case report

**DOI:** 10.1186/cc3034

**Published:** 2005-01-21

**Authors:** Toshio Naka, Daryl Jones, Ian Baldwin, Nigel Fealy, Samantha Bates, Hermann Goehl, Stanislao Morgera, Hans H Neumayer, Rinaldo Bellomo

**Affiliations:** 1 Research Fellow, Department of Intensive Care and Department of Medicine, Melbourne University, Austin Hospital, Melbourne, Australia; 2 Registrar, Department of Intensive Care and Department of Medicine, Melbourne University, Austin Hospital, Melbourne, Australia; 3 Nurse Educator, Department of Intensive Care and Department of Medicine, Melbourne University, Austin Hospital, Melbourne, Australia and PhD Candidate, Latrobe University, Bundoora, Melbourne, Australia; 4 Nurse Educator, Department of Intensive Care and Department of Medicine, Melbourne University, Austin Hospital, Melbourne, Australia; 5 Research Nurse, Department of Intensive Care and Department of Medicine, Melbourne University, Austin Hospital, Melbourne, Australia; 6 Chief Engineer, Gambro Dialysatoren GmbH & Co., KG, Hechingen, Germany; 7 Staff Specialist, Department of Nephrology, Charitè, Humboldt, University of Berlin, Germany; 8 Director, Department of Nephrology, Charitè, Humboldt, University of Berlin, Germany; 9 Director of Research, Department of Intensive Care and Department of Medicine, Melbourne University, Austin Hospital, Melbourne, Australia

## Abstract

**Objective:**

To test the ability of a novel super high-flux (SHF) membrane with a larger pore size to clear myoglobin from serum.

**Setting:**

The intensive care unit of a university teaching hospital.

**Subject:**

A patient with serotonin syndrome complicated by severe rhabodomyolysis and oliguric acute renal failure

**Method:**

Initially continuous veno-venous hemofiltration was performed at 2 l/hour ultrafiltration (UF) with a standard polysulphone 1.4 m^2 ^membrane (cutoff point, 20 kDa), followed by continuous veno-venous hemofiltration with a SHF membrane (cutoff point, 100 kDa) at 2 l/hour UF, then at 3 l/hour UF and then at 4 l/hour UF, in an attempt to clear myoglobin.

**Results:**

The myoglobin concentration in the ultrafiltrate at 2 l/hour exchange was at least five times greater with the SHF membrane than with the conventional membrane (>100,000 μg/l versus 23,003 μg/l). The sieving coefficients with the SHF membrane at 3 l/hour UF and 4 l/hour UF were 72.2% and 68.8%, respectively. The amount of myoglobin removed with the conventional membrane was 1.1 g/day compared with 4.4–5.1 g/day for the SHF membrane. The SHF membrane achieved a clearance of up to 56.4 l/day, and achieved a reduction in serum myoglobin concentration from >100,000 μg/l to 16,542 μg/l in 48 hours.

**Conclusions:**

SHF hemofiltration achieved a much greater clearance of myoglobin than conventional hemofiltration, and it may provide a potential modality for the treatment of myoglobinuric acute renal failure.

## Introduction

Rhabdomyolysis and myoglobinuria are responsible for approximately 5% of all causes of acute renal failure (ARF) in the USA [1]. The cause of rhabdomyolysis is often multifactorial [2], and approximately 8–20% of such patients develop myoglobinuric ARF [3]. The myoglobin released during rhabdomyolysis is thought to cause renal dysfunction by producing renal tubular obstruction, lipid peroxidation within the tubular cells, and renal vasoconstriction [1]. Intravascular volume expansion, urinary alkalinization, and forced diuresis are currently used as renal-protective measures [4]. These treatments are not useful in the context of severe oliguria, and may also cause pulmonary edema. In the setting of oliguria, removal of myoglobin using blood purification techniques may be advantageous.

Previous attempts to remove myoglobin using plasma exchange [5,6], intermittent hemodialysis [7], and continuous renal replacement therapies have unfortunately met with limited success [6-11].

We have recently described a novel super high-flux (SHF) membrane with a pore cutoff size of 100 kDa, which has the ability to dialyze inflammatory cytokines and β_2_-microglobulin [12-15]. We now describe the use of SHF hemofiltration in a patient with severe rhabdomyolysis and oliguric ARF secondary to serotonin syndrome, and report on the kinetics of myoglobin clearance using both conventional and SHF hemofiltration.

## Case report

A 53-year-old female presented with complications of a polypharmacy overdose. Her past medical history included depression, hyperlipidemia, and anorexia nervosa. There was a previous history of amphetamine abuse as well as two prior episodes of polypharmacy overdose. The patient was discovered at home with a Glasgow Coma Score of 11 and was noted to be hypotensive and tachycardic. Soon after transfer to hospital, the patient became unconscious (Glasgow Coma Score, 3) and was intubated. Empty bottles of paroxetine, moclobemide, pravastatin, and alprazolam were found in her house. The patient was not known to be on neuroleptic antipsychotic medications, and had not used amphetamine for at least 1 year.

The patient subsequently developed features of serotonin syndrome [16,17], with progressive hypotension despite a central venous pressure of 10 mmHg and commencement of norepinephrine to a maximum dose of 2.5 μg/kg/min, as well as 10 l fluid resuscitation on the first day of admission. Additional features of serotonin syndrome included fever (42°C) and generalized hypertonicity, which was complicated by severe rhabdomyolyis (peak creatine kinase level, 109,000 IU/l). Furthermore, there was evidence of coagulopathy, with severe bleeding from puncture sites and the gastrointestinal tract. Investigations confirmed the presence of disseminated intravascular coagulopathy.

The patient developed profound metabolic derangement with lactic acidemia (lactate peak, 11.7 mmol/l; pH nadir, 7.14), oliguric ARF, severe hyperkalemia (K^+^, 9.1 mmol/l at presentation), hypocalcemia, hyperphosphatemia, and hypomagnesemia, and with the subsequent development of ischemic hepatitis (alanine aminotransferase, 4048 U/l; alkaline phosphatase, 92 U/l). On the first intensive care unit day, continuous veno-venous hemofiltration (CVVH) with a conventional hemofilter was employed for the management of oliguric ARF and hyperkalemia.

Treatment with fresh frozen plasma, cryoprecipitate, platelet and red blood cells was required. Specific treatment of serotonin syndrome was based on therapies described elsewhere [16,17], and included active cooling, cyproheptadine, a midazolam infusion, and neuromuscular blockade with vecuronium.

## Conventional CVVH and SHF CVVH

A 13 Fr dual-lumen catheter (Niagara, Bard, Toronto, Canada) was inserted into a femoral vein and CVVH was performed using a Kimal Hygieia Plus machine (Medtel, Perth, Australia). The blood flow was set at 200 ml/min and the ultrafiltration (UF) rate was set at 2 l/hour. A bicarbonate-based commercial replacement fluid (Hemosol; Gambro, Sydney, Australia) was administered at the pre-filter site. Initially a polysulphone 1.4 m^2 ^membrane (molecular cutoff, 20 kDa) (APS; ASAHI-medical, Tokyo, Japan) was used for conventional CVVH. On the following day, the hemofilter was changed to a novel SHF membrane (molecular cutoff point, 100 kDa) (Polyflux P2SH; Gambro Dialysatoren, Hechingen, Germany). The UF volume rate was commenced at 2 l/hour and sequentially increased to 4 l/hour over an 8-hour period, before being maintained at 2 l/hour.

## Measurement and calculations

Blood samples were collected at pre-filter and post-filter sites, and the serum was separated immediately. Ultrafiltrate was collected simultaneously and all specimens were frozen at -70°C until measurements were performed. Myoglobin was measured by a microparticle enzyme immunoassay (AxSYM system; Abbot Laboratories, Perth, Australia). Using this AxSYM system assay, the normal lower limit for serum myoglobin is 150 μg/l and the upper limit of testing is 100,000 μg/l.

The sieving coefficient (SC) was calculated using the formula: SC = 2*C*_uf_/(*C*_pre _+ *C*_post_), where *C*_uf _represents the concentration of myoglobin in the ultrafiltrate, and *C*_pre _and *C*_post _are the pre-filter and post-filter concentrations of myoglobin, respectively.

The amount of removed myoglobin was calculated as *C*_uf _× *V*_uf_, where *C*_uf _represents the concentration of myoglobin in the ultrafiltrate and *V*_uf _represents the UF volume per unit of time. The clearance of myoglobin was calculated as *C*_uf _× *V*_uf_/*C*_pre_.

## Results

The serum myoglobin concentration was >100,000 μg/l at the initiation of conventional CVVH, and remained so at commencement of SHF CVVH on the following day (Table 1). The myoglobin concentration in the ultrafiltrate at 2 l/hour UF exchange was at least five times greater with the SHF membrane than with the conventional membrane (>100,000 μg/l versus 23,003 μg/l).

**Figure 1:**
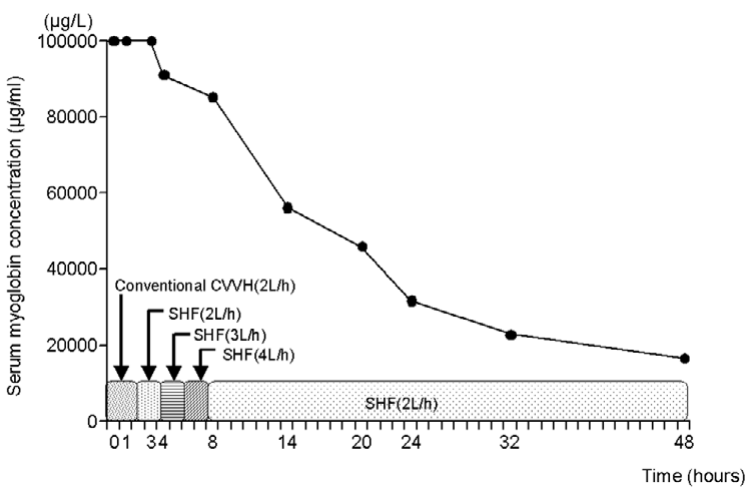
Progressive reduction of serum myoglobin concentration over a 48-hour period using super high-flux (SHF) continuous veno-venous hemofiltration (CVVH). The serum concentration of myoglobin remained >100,000 μg/l until commencement of SHF CVVH.

**Figure 2:**
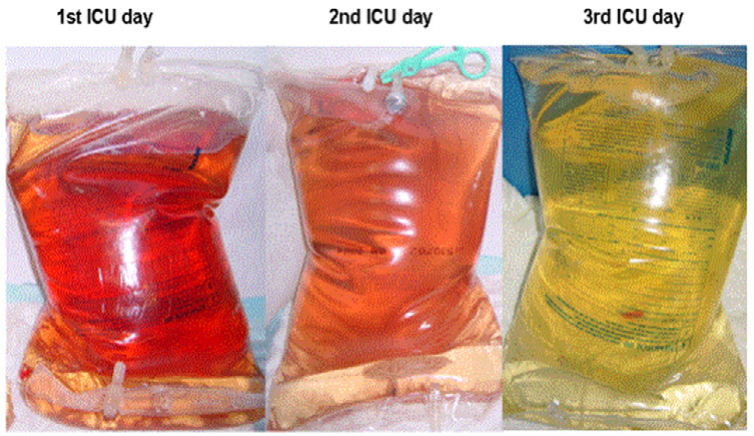
Images of ultrafiltration fluid demonstrating progressive reduction in pigmentation over a 3-day period concurrent with treatment using super high-flux continuous veno-venous hemofiltration. ICU, intensive care unit.

The SCs with SHF hemofiltration at 3 l/hour UF and 4 l/hour UF exchange rates were 72.2% and 68.8%, respectively, resulting in the removal of 4.3–5.1 g myoglobin/day and in myoglobin clearances of up to 56.4 l/day. Treatment with SHF hemofiltration resulted in a reduction of the serum myoglobin concentration from >100,000 μg/l to 16,542 μg/l in 48 hours (Fig. 1). There was a concurrent reduction in the degree of pigmentation of the UF fluid over this period (Fig. 2).

## Discussion

In the case described in the present study, SHF CVVH achieved myoglobin clearance values significantly greater than control treatment with standard high-flux hemofiltration, and values greater than those previously reported in the literature [8-11]. Clearance of myoglobin using SHF CVVH was greater than the total body water over a 24-hour period.

Multiple modalities of renal replacement therapy have been used in the past in an attempt to clear myoglobin. However, these modalities have shown limited efficiency [8-11] (Table 2). We have previously presented the results of myoglobin clearances in three critically ill patients with rhabdomyolysis treated with continuous hemodiafiltration, which was associated with a mean daily myoglobin removal of 1.8 g/day with a myoglobin clearance of 4.6 ml/min [8].

Continuous arterio-venous hemofiltration and CVVH have been used to investigate myoglobin clearance in a swine model of myoglobinuric ARF. The use of continuous arterio-venous hemofiltration and a Hospal AN 69S hemofilter resulted in myoglobin clearances of 2.05 ± 1.48 l/day, with 410 ± 234 mg myoglobin excreted in the ultrafiltrate over the 6-hour period [9].

CVVH has more recently been used in a single case of myoglobin-induced renal failure [11]. Using a Hospal AN69 Multiflow-100 hemofilter, the SC for myoglobin was reported at 40–60%. The corresponding clearance of myoglobin over this period was reported to be up to 22 ml/min (32 l/day).

In the present study, the use of CVVH with a novel SHF hemofilter and UF rates of 3–4 l/min was associated with a SC of 69–72% and a clearance of up to 56.4 l/day. This was approximately five times greater than the clearance achieved with conventional hemofiltration (control) in the same patient. These results are in keeping with the known difference in the pore size of the two filters studied (polysulfone cutoff point, 20 kDa versus polyamide cut-off point, 100 kDa) in relation to the molecular weight of myoglobin (17 kDa), and are also consistent with observations that SHF CVVH can achieve high clearances of other middle molecules such as β_2_-microglobulin and cytokines [12-15].

Two previous studies utilizing CVVH and high-flux hemofiltration membranes have documented 24-hour myoglobin clearances of approximately 4.3 g [18,19]. The SC using a Gambro polyflux 11S membrane was only 37%, however, and neither of these studies reported on myoglobin clearance or the cutoff size of the pores of the membrane used.

A potential adverse effect of SHF (high cutoff point) hemofiltration is the loss of serum albumin (69 kDa). The SC for albumin with SHF hemofiltration has been shown to be at 0.11 at 1 l/hour exchange [12], so that a patient with a plasma albumin concentration of 20 g/l would be expected to lose 2.2 g/hour albumin. Over a 24-hour treatment period, this might necessitate albumin replacement using 200 ml of 20% albumin. Our patient received volume expansion with albumin (100 g over a 24-hour period) as part of her treatment, and her albumin level increased from 21 to 32 g/l (Table 3) despite SHF hemofiltration. Nonetheless, there is a possibility for losses of albumin and, perhaps, for losses of clotting factors or protein-bound drugs such as fentanyl and midazolam. We thus consider that monitoring of albumin levels and the clotting status, and adjustment of the drug dosage might be important when treating patients with SHF hemofiltration.

## Conclusion

The current case offers 'proof of concept' that SHF CVVH can be employed to clear myoglobin effectively in patients with rhabdomolysis and ARF. The technique may be of greatest advantage in patients with oliguric renal failure and in those already requiring renal replacement therapy. It remains to be determined whether this technique is capable of altering renal recovery or mortality in patients with rhabdomyolysis and myoglobinuria-related ARF.

## Key messages

• 	New super high-flux membranes when used in hemofiltration mode can achieve the highest clearances ever reported for myoglobin and may be useful in patients with severe rhabdomyolysis

• 	New super high-flux membranes when used in hemofiltration result in some albumin losses. These losses, however, decrease in time and are not vary large

## Abbreviations

ARF = acute renal failure; CVVH = continuous veno-venous hemofiltration; SC = sieving coefficient; SHF = super high-flux; UF = ultrafiltration.

## Competing interests

The author(s) declare that they have no competing interests.

## Authors' contributions

TN, DJ, IB, NF, and SB conducted the study and collected the data, and helped write the paper. HG, SM, HHN, and RB helped develop and test the membrane, and also helped write the paper.

## Figures and Tables

**Table 1:** Sieving coefficient and clearance of myoglobin using conventional and super high-flux (SHF) continuous veno-venous hemofiltration in a patient with severe rhabdomyolysis secondary to serotonin syndrome

Filter type	Ultrafiltration rate (l/hour)	Myoglobin concentration (μg/l)			Sieving coefficient (%)	Myoglobin removal (g/day)	Myoglobin clearance (ml/min)
					
		Pre-filter	Post-filter	Ultrafiltrate			
Conventional (polysulphone)	2	>100,000	>100,000	23003	<0.23	1.1	<8
SHF	2	>100,000	>100,000	>100,000	Unable to calculate	>4.8	Unable to calculate
SHF	3	100,000	68,776	60,912	0.722	4.4	30.5
SHF	4	91,058	64,587	53,527	0.688	5.1	39.2

**Table 2:** Comparison of studies of renal replacement therapy (RRT) in the management of myoglobinuric acute renal failure

Reference	Subject(s)	Mode of RRT	Filter used	Sieving coefficient (%)	Myoglobin removal (g/day)	Clearance (ml/min)
Present study	Single patient with ARF due to rhabdomyolysis	CVVH, 2 l exchange	Polysulphone (APS)	<0.23	1.1	<8
		SHF CVVH, 3–4 l exchange	Gambro Polyflux P2SH	0.69–0.72	4.3–5.1	30.5–39.2
[8]	Three patients with myoglobinuric ARF	Continuous hemodiafiltration	Hospal AN 69S	0.21*	1.8	4.6
[9]	Swine model of myoglobinuric ARF	CAVH	Hospal AN 69S	0.15	1.64 (0.41 g/6 hours)	1.42 ± 1.03
[10]	Swine model	CVVH	Hospal AN 69HF	0.36–0.55	2.3–3.8 (0.95 g/6 hours)	11.2–25.2
[11]	Single patient with ARF due to rhabdomyolysis	CVVH	Hospal AN69 Multiflow 100	0.40–0.60	1.05 (0.7 g/16 hours)	14–22
[19]	Patient with malignant hyperthermia and rhabdomyolysis	CVVH	Gambro polyflux 11S	0.37	4.3	11*

ARF, acute renal failure; CAVH, continuous arterio-venous haemofiltration; CVVH, continuous veno-venous haemofiltration; SHF, super high-flux. * Calculated from the *C*_UF_, *V*_UF_, and *C*_pre _values provided in these studies

**Table 3:** Summary of laboratory investigations and blood product administration

	Day 1	Day 2	Day 3	Day 4	Day 5
Na^+^	144	144	137	131	129
K^+^	9.1	4.2	3.9	4.4	4.6
Cl^-^	109	107	98	99	95
HCO_3_	20	18	19	19	13
Urea	9	10.3	6.5	4.3	3.7
Creatinine	0.127	0.214	0.111	0.118	0.107
Ca^2+^	1.64	1.13	2.3	2.25	2.26
PO_4_	2.1	3.22	1.8	1.24	1.6
Albumin	21	29	32	29	31
Creatine kinase	5199	90,000	71,120	85,426	87,170
Lactate	6.42	9.13	7.37	8.02	13.21
Hemoglobin	106	8.6	82	86	73
White cell count	4.8	5	2	4.7	2.5
Platelets	173	72	31	64	112
International normalized ratio for prothrombin time	1.5	4.6	1.9	3.5	5.4
Activated partial thromboplastin time	32	143	44	64	83
Packed red blood cell units	2	3	0	0	0
Platelets	5	5	5	0	0
Fresh frozen plasma	0	26	2	5	0
Cryoprecipitate	10	14	3	0	0
Albumin (20% solution) (ml)	200	500	100	200	0
Norepinephrine dose (μg/min)	3	6	25	30	100
